# Atomic Layered ZnO Between Cu Nanoparticles and a PVP Polymer Layer Enable Exceptional Selectivity and Stability in Electrocatalytic CO_2_ Reduction to C_2_H_4_

**DOI:** 10.1002/advs.202501642

**Published:** 2025-04-26

**Authors:** Lihui Zhou, Hung‐Wei Tsai, Ting‐Wei Kuo, Jui‐Cheng Kao, Yu‐Chieh Lo, Ji‐Min Chang, Tzu‐Hsuan Chiang, Sheng Dai, Kuan‐Wen Wang, Tsan‐Yao Chen

**Affiliations:** ^1^ Key Laboratory for Advanced Materials and Feringa Nobel Prize Scientist Joint Research Centre School of Chemistry and Molecular Engineering East China University of Science & Technology Shanghai 200237 China; ^2^ Institute of Materials Science and Engineering National Central University Taoyuan 32001 Taiwan; ^3^ Department of Materials Science and Engineering National Yang Ming Chiao Tung University Hsinchu 30010 Taiwan; ^4^ Department of Energy Engineering National United University Miaoli 360301 Taiwan; ^5^ Department of Engineering and System Science National Tsing Hua University Hsinchu 30013 Taiwan; ^6^ Institute of Analytical and Environmental Science National Tsing Hua University Hsinchu 30013 Taiwan; ^7^ Institute of Nuclear Engineering and Science National Tsing Hua University Hsinchu 30013 Taiwan

**Keywords:** atomic oxide layers, CO2 electrochemical reduction, Cu nanoparticles, PVP polymer

## Abstract

This study employs a chemically controlled strategy to construct a few‐atomic‐layer ZnO structure integrated with polyvinylpyrrolidone (PVP) and nanoscale metallic copper on active carbon. Hydrogen‐bond interactions from PVP's N‐vinylpyrrolidone allow ZnO to retain a specific proportion of metal atoms, confining electrons at the Cu/ZnO interface to form CuZn nanoalloy clusters. The nanoalloy's dual role in promoting CO adsorption and C─C coupling synergistically boosts C_2_H_4_ production during electrochemical CO_2_ reduction (ECR). Rapid Cu regeneration further increases adsorbed hydrogen (H^ads^) from water splitting, achieving a remarkable C_2_H_4_ selectivity of ≈50.2% with stable performance over 10 h. The Zn→Cu electron confinement and interfacial synergy at the organic‐oxide‐metal heterojunction underscore the catalyst's superior efficiency, offering a promising pathway for sustainable CO_2_‐to‐C_2_H_4_ conversion.

## Graphical abstract

Collaboration between Cu, ZnO, and PVP facilitates the electrochemical reduction of CO_2_.

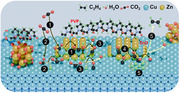

## 1 Introduction

Electrochemical catalysis has emerged as one of the most pivotal and efficient strategies for the conversion of carbon dioxide (CO₂) into valuable hydrocarbon molecules, particularly alkanes. This technology holds immense promise for mitigating environmental carbon emissions by facilitating the transformation of CO₂ into energy‐dense products under sustainable conditions. Among the diverse classes of heterogeneous catalysts, copper‐based metallic nanoparticles (Cu NPs) have garnered significant attention for their exceptional performance in the electrochemical CO₂ reduction reaction (ECR). The remarkable catalytic performance is primarily attributed to its unique surface chemistry. Copper atoms on the catalyst surface exhibit high intrinsic activity, enabling the effective dissociation of CO₂ molecules under electrochemical conditions.^[^ ^1^ ^]^ Furthermore, copper's moderate adsorption energy (E^ads^) for chemisorbed CO intermediates (CO^ads^) distinguishes it from other metals and metal oxides, as this property prolongs the residence time of CO^ads^ on the catalytic surface.^[^ ^2^ ^]^ This extended interaction enhances the likelihood of subsequent reactions with hydrogen atoms (H^ads^) generated via water dissociation in the neighboring sites, thereby facilitating the formation of multi‐carbon products through C─C coupling mechanisms. Despite the promising activity and selectivity of monometallic Cu NPs in ECR, their long‐term chemical stability remains a critical challenge. The durability of Cu‐based catalysts diminishes over time, predominantly due to structural and compositional transformations induced by complex redox reaction pathways.^[^ ^3^ ^]^ Key degradation mechanisms include 1. oxidation of surface‐active sites, 2. nanoparticle aggregation, and 3. morphological changes under electrochemical operating conditions.

1. Oxidation of surface‐active sites: Copper intrinsically tends to oxidize, forming a CuO layer that deactivates the surface and reduces reactivity during prolonged ECR:^[^ ^4^, ^5^ ^]^ As the oxide layer accumulates, the reactive surface area for adsorption and decomposition of reactants decreases, resulting in a decline in reaction current and operational lifespan.
2. Nanoparticle aggregation: During ECR, Cu NP dissolution forms ions that redeposit on larger particles, reducing smaller ones. This decreases the active surface area and lowers reaction current, despite constant material mass.^[^ ^6^ ^]^
3. Morphological Changes Under Electrochemical Operating Conditions: Atomic spacing and symmetry differences affect adsorption energies and reaction rates, with ECR‐induced energy causing nanoparticle surface atoms to migrate, reducing high‐energy facets and catalytic activity.^[^ ^7^, ^8^, ^9^, ^10^ ^]^

To mitigate atomic diffusion and surface oxidation that degrade the efficiency of metallic nanoparticles (NPs) during prolonged ECR, scientists have developed advanced chemical processing techniques. These methods precisely control reaction pathways, reactant concentrations, and temperatures while using small molecule ligands to chelate metal ions, optimizing both surface and interior atomic structures of NPs.^[^ ^11^ ^]^ Key innovations include stabilizing Cu‐based NPs through homogeneous alloying with heterogeneous metals (M) or creating Cu_core_‐M_shell_ architectures.^[^ ^12^, ^13^, ^14^ ^]^ These designs promote synergistic interactions, enhancing bifunctional mechanisms, ligand effects, and interfacial lattice strain,^[^ ^15^ ^]^ collectively lowering CO^ads^ adsorption energy (E^ads^‐CO).^[^ ^11^, ^16^ ^]^ This enables higher yields of valuable products like CO and C₂H₄. Another breakthrough is the use of organic porous frameworks, such as ZIF‐8 or BIF, which allow Cu atoms to form unique nanogeometries, unattainable with traditional supports.^[^ ^17^, ^18^ ^]^ These structures alter morphology and establish distinct local chemical environments, enhancing catalyst stability during ECR. Additionally, doping Cu NPs with heterogeneous elements like Zn, Pd, or Al modifies their electronic structures, further reducing E^ads^‐CO and optimizing CO desorption. For example, Al or Zn doping in Cu₂O strengthens structural integrity and increases C₂⁺ hydrocarbon yields. These strategies represent a paradigm shift in catalyst design, integrating structural reinforcement with enhanced product selectivity. By combining advanced synthesis methods with insights into catalytic mechanisms, they pave the way for robust, efficient, and sustainable CO₂ conversion technologies.^[^ ^19^, ^20^ ^]^

Despite progress, challenges like material corrosion and oxidation hinder long‐term stability for high‐carbon product generation. Advanced strategies now focus on integrating heterogeneous active sites through heteroatom doping or ligand adsorption. Coupling In₂O clusters with Ag nanoparticles creates synergistic interfaces between oxygen vacancies O^V^s and metal atoms, significantly enhancing formic acid yields.^[^ ^21^ ^]^ A trace amount of In_2_O_3_ decorated on Cu surface greatly improves the selectivity and stability for CO_2_‐to‐CO reduction.^[^ ^22^ ^]^ Decorating AuCu clusters on Cu nanoparticles improves CO selectivity and catalyst lifespan.^[^ ^23^ ^]^ In this work, we introduced a Zn oxide atomic layer to bridge hydrogen‐bonding polymer chains (polyvidone ligand) with Cu NPs, forming a robust CuZnO‐PVP nanocomposite. PVP has been reported to function as both a steric stabilizer^[^ ^24^ ^]^ and an electronic modifier,^[^ ^25^ ^]^ enabling precise control over catalyst performance in CO₂RR. The interaction between PVP's pyrrolidone groups and metal surfaces modulates charge distribution, enhances CO₂ adsorption, and suppresses the hydrogen evolution reaction (HER).^[^ ^24^, ^26^ ^]^ On the other hand, previously, we found that Zn significantly mitigates metal atom mobility, enhances dissolution resistance, and improves both structural and performance stability while maintaining excellent CO₂‐to‐CO conversion efficiency.^[^ ^27^ ^]^ Additionally, ZnO donates electrons to Ag, facilitating the adsorption and structural rearrangement of CO₂ molecules, followed by the desorption of CO.^[^ ^28^ ^]^ Here, this organic‐oxide‐metal interface facilitates distinct functionalities: The polyvidone ligand preferentially binds to ZnO, weakening O^ads^ diffusion to the Cu‐ZnO interface, thus preventing stable oxide formation and preserving active sites. Additionally, the ligand's strong affinity for ZnO enhances CO₂ accumulation on Cu, increasing reactant concentrations and significantly boosting C₂H₄ production.^[^ ^29^ ^]^ The Zn atomic layer, stabilized by PVP's polarization effects, further inhibits Cu oxidation. These synergistic interactions enable exceptional durability under high‐current ECR conditions. Besides, a structure‐activity relationship specific to triple‐phase interfaces is established, addressing atomic‐scale organic‐inorganic co‐modification, in situ spectroscopic analyses (XAS & Raman), and ligand‐metal‐oxide interplay. These synergistic interactions within the triphasic interface enable exceptional catalytic durability under high‐current ECR conditions, highlighting a transformative approach to CO₂ reduction technologies. It is worth mentioning that compared to traditional H‐type cells, the thermodynamics and kinetics of ECR in flow cells are fundamentally different, making them a more promising approach for large‐scale commercial applications. However, H‐type cells serve as suitable batch reactors for laboratory‐scale quantification and selection of electrocatalysts for CO_2_ reduction to various products.^[^ ^30^ ^]^ Therefore, in this study, ECR and in situ spectroscopic investigations of Cu‐based catalysts are conducted in an H‐type cell.

## 2 Results and Discussion

### 2.1 Physical Structure Inspections

**Figure 1:**
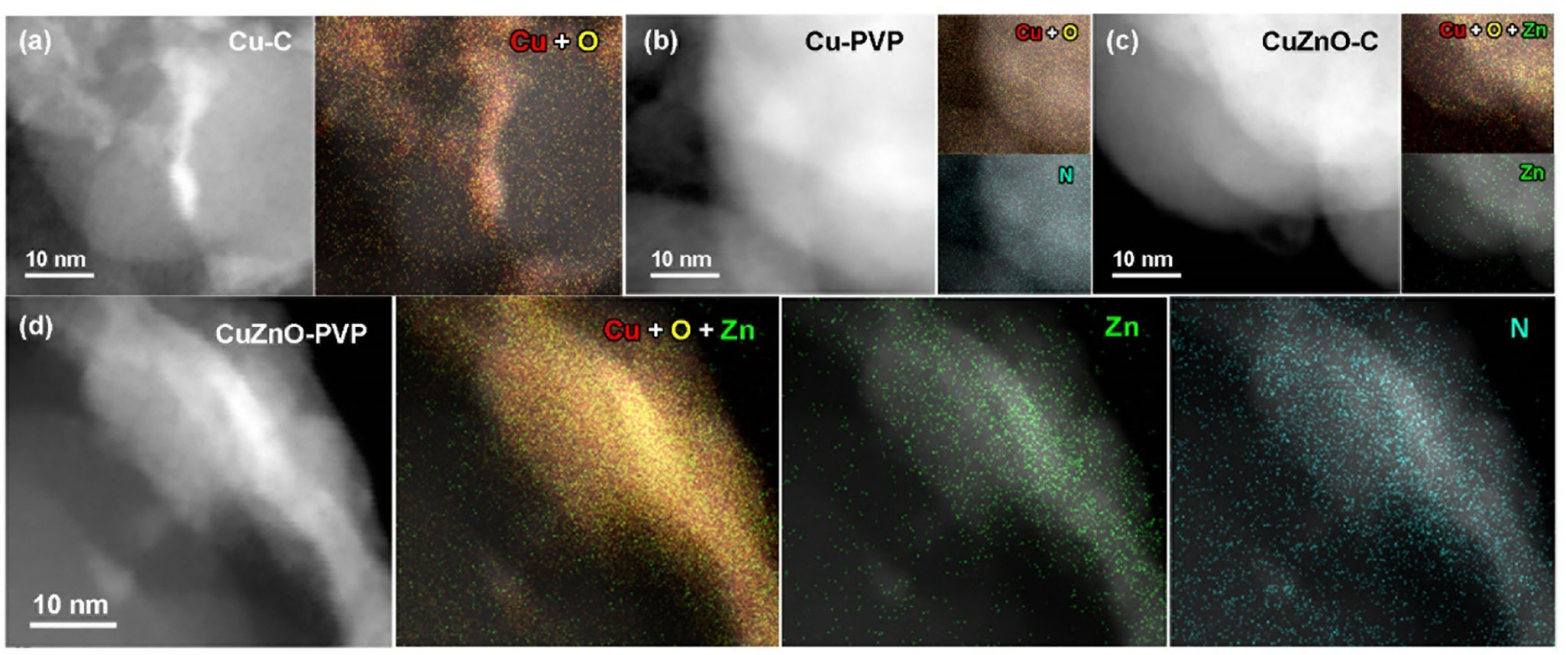
HAADF‐STEM characterization and corresponding EDS elemental maps of various Cu‐based catalysts. a) Cu‐C, b) Cu‐PVP, c) CuZnO‐C, and d) CuZnO‐PVP.

**Figure** **1** presents HAADF‐STEM images and EDS mappings of carbon‐supported Cu and CuZnO nanoparticles, both with (Cu‐PVP and CuZnO‐PVP) and without PVP (Cu─C and CuZnO─C). To further elucidate the nanostructure, the corresponding X‐ray powder diffraction (XRD) patterns are provided in Figure S1a (supporting information), with structural parameters summarized in Figure S1b and Table S1 (Supporting Information). From the HAADF images, it is evident that in the Cu‐C sample, copper atoms form nanoscale clusters on the activated carbon surface. The EDS mapping shows significant overlap between copper and oxygen, suggesting that Cu primarily exists as metal oxides. In the Cu‐PVP sample, Cu atoms are uniformly distributed on the activated carbon surface (Figure 1b), with nitrogen distribution confirming the even presence of polyvinylpyrrolidone (PVP) on the sample. In the CuZnO─C sample, Cu atoms also form a uniform nanostructure on activated carbon, similar to Cu‐C. However, the low contrast and density of Zn in the EDS mapping indicate that a very thin ZnO layer coats the Cu NPs. For the CuZnO‐PVP sample, the EDS mappings show overlapping distributions of Cu, Zn, and N, indicating that the nanostructures are coated with PVP molecules. Notably, the Zn and N overlaps with the HAADF image, confirming that Zn is localized on the Cu nanostructure surface, with some Cu atoms remaining exposed. The PVP polymer is uniformly distributed, covering both the Cu NPs and the ZnO thin film. XRD analysis (Figure S1, Supporting Information) estimates the coherent lengths (D) of the copper and ZnO nanostructures to be 15–42 and 22–23 nm, respectively. However, these values are larger than the spatial scale of Cu and Zn distributions observed in the STEM/EDS mappings, indicating that the XRD diffraction peaks correspond to larger crystallites, not the full range of Cu and Zn nanostructures.

### 2.2 Atomic and Electronic Structure Analysis

**Figure 2:**
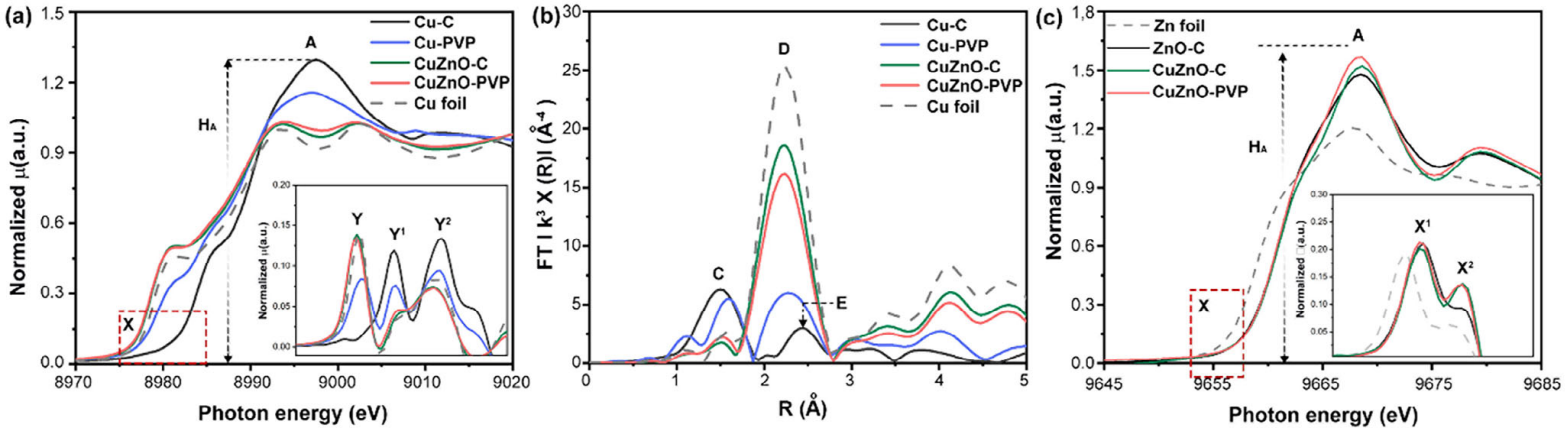
X‐ray absorption spectroscopy of the experimental samples compared with reference samples. a) XANES and b) FT‐EXAFS spectra of the experimental samples at the Cu K‐edge. c) XANES spectra of the experimental samples at the Zn K‐edge. The first derivative curves of XANES at Cu and Zn K‐edge have been shown in the inset.

**Figure** **2**a compares the X‐ray absorption near‐edge structure (XANES) spectra of experimental samples (Cu─C, Cu─PVP, CuZnO─C, and CuZnO‐PVP) with the reference Cu foil. The area under the white line (WL) or the intensity of absorption peak A (H_A_) corresponds to the density of unoccupied d‐states, making it sensitive to the oxidation state and changes in the electronic structure due to chemical bonding or charge transfer phenomena.^[^ ^31^ ^]^ In Figure 2a, the H_A_ of Cu‐C decreases upon ZnO addition, suggesting that ZnO either protects Cu from oxidation or facilitates electron transfer from Zn to Cu. In the pre‐edge region (X), the position of the inflection point (peak Y) and the profile of the first derivative curve (Figure 2a inset) reflect the oxidation state of Cu and the degree of hybridization of its 4s/4p orbitals with neighboring atoms. Extended X‐ray absorption fine structure (EXAFS) spectra and quantitative model analyses are presented in Figure 2b and **Table** **1** for further structural interpretation. Compared to Cu foil, the Cu‐C sample shows a significant shift in the inflection point energy (from Y to Y^1^ in the inset) and a higher intensity of H_A_, indicating pronounced Cu oxidation. EXAFS analysis reveals Cu oxide contributions with radial peaks C (Cu─O at 1.947 Å, CN = 4.35) and E (Cu─Cu/Cu─O bond pairs at the 2nd coordination shell of Cu_2_O 1.49/1.86 Å) within Cu‐C (Table 1). For Cu‐PVP, the shift of the inflection point (from Y to Y^2^) and the splitting of peak Y^2^ (into Y^2^ and Y^1^) suggest partial protection of Cu from oxidation due to the steric shielding of the PVP capping layer. Despite this, the low affinity of the pyrrolidone ligand leads to incomplete protection, resulting in partial oxidation and the coexistence of Cu oxide (radial peak C, CN = 2.65 for Cu─O) and metallic Cu (radial peak E, CN = 2.65 for Cu─Cu). In contrast, Zn oxide provides stronger surface protection for Cu, maintaining the Cu NPs in a metallic state, as confirmed by XANES and EXAFS spectra for CuZnO‐C. The XANES spectrum of CuZnO‐C closely resembles that of Cu foil, indicating that Cu atoms remain metallic. EXAFS reveals the coexistence of Cu‐Cu and Cu─Zn bonds (CN = 8.01 and 0.89, respectively), suggesting slight Zn incorporation on the Cu NP surface, as seen in the near‐edge spectrum feature (Figure 2a inset).

**Table 1:** The model analysis determined structure parameters of Cu─C, Cu‐PVP, CuZnO‐C, and CuZnO‐PVP at Cu K‐edge.

Sample	CN				R (Å)			
	Cu─O^ads^	Cu─O	Cu─Cu^M^	Cu─Zn^M^	Cu─O^ads^	Cu─O	Cu─Cu^M^	Cu─Zn^M^
Cu─C	4.35	1.86^*)^	1.49^*)^	N/A	1.947	2.997^*)^	2.929^*)^	N/A
Cu‐PVP	2.65	N/A	2.65	N/A	1.983	N/A	2.592	N/A
CuZnO─C	0.34	N/A	8.01	0.89	1.846	N/A	2.533	2.533
CuZnO‐PVP	0.53	N/A	6.68	0.88	1.877	N/A	2.533	2.533

*) denote the bond paths of Cu‐O and Cu‐Cu in the 2nd coordination shell of Cu2O.

Figure 2c shows the Zn K‐edge XANES spectra of experimental samples (CuZnO‐C and CuZnO‐PVP), the control sample (ZnO‐C), and the reference sample (Zn foil). The first derivative spectrum in the near‐edge region (region X) is shown as an inset. Compared to the Zn foil, the energy of the near‐edge inflection points X^1^ and X^2^, which are identical to those in the ZnO spectrum, indicates that the oxidation state of Zn atoms in CuZnO‐C and CuZnO‐PVP is the same as in ZnO. Meanwhile, the significant increase in intensity at position X^2^ suggests that, in addition to bonding with oxygen atoms, Zn atoms in the samples also exhibit heteroatomic hybridization with other metal atoms (i.e., Cu in this study). Moreover, since Zn atoms in all samples share the same oxidation state, the increased H_A_ intensity of CuZnO─C relative to ZnO─C indicates a greater number of empty states in the 4s/4p orbitals, confirming that Zn donates electrons to Cu. This effect is further enhanced in the presence of PVP molecules, which make Zn atoms more likely to transfer electrons to surrounding Cu atoms. The aforementioned effects modify the chemical properties of Zn atoms, enhancing their CO adsorption. This is confirmed by the CO‐stripping results in Figure S2 (Supporting Information), where the CO‐stripping peak becomes more pronounced after ZnO addition, indicating its increased ability to adsorb CO. Moreover, this CO adsorption behavior and the *CO intermediate have been investigated using in situ Raman spectroscopy. As shown in Figure S3 (Supporting Information), an absorption band centered at ∼2080 cm⁻¹ appears at cathodic potentials of −0.2 and −0.6 V for CuZnO‐PVP and CuZnO─C catalysts, respectively. This band, typically attributed to the C≡O stretching of atop‐adsorbed *CO species,^[^ ^32^ ^]^ is absent in Cu─C, indicating that both ZnO and PVP facilitate CO adsorption and enhance *CO coverage from activated CO₂, particularly in CuZnO‐PVP. This enables a longer interaction time with Cu atoms during the ECR process, where hydrogen atoms are provided, resulting in the production of high‐carbon hydrocarbons. Complimentary evidences are given by X‐ray photoemission spectroscopy (XPS) analysis in supporting information. Figure S4 (Supporting Information) demonstrates the XPS spectra of experimental samples (Cu─C, Cu‐PVP, CuZnO‐C, and CuZnO‐PVP) and the control sample (ZnO─C) at the Cu 2p, Zn 2p, and N 1s orbitals. The quantitative analysis results of the spectra are summarized in Table S2 (Supporting Information). Accordingly, the surface composition of Cu‐C consists of 25% Cu^2^⁺ and 75% Cu⁰. Compared to Cu─C, the Cu‐PVP sample shows a 4% increase in the surface metal ratio, confirming that PVP molecules indeed exhibit a steric protection effect on Cu atoms. The surface oxidation composition of Cu atoms in the CuZnO‐C sample is identical to that of Cu‐PVP, while the surface Cu/Zn atomic ratio is 68/32. Given that the overall Cu/Zn ratio of the sample is 93/7, this result indicates that Zn atoms preferentially segregate to the surface of Cu NPs, leading to significant heteroatomic intermixing effects of Cu and Zn, consistent with the analysis of the first derivative in Zn XANES (Figure 2c). Compared to CuZnO‐C, the oxidation state of Cu atoms on the surface of CuZnO‐PVP increases by 28%. With a similar Cu/Zn compositional ratio, this result suggests that the functional groups in PVP molecules tend to bond with Zn atoms, reducing the steric protection effect on Cu atoms. This finding is consistent with the analysis results from the Cu K‐edge XAS spectrum. Furthermore, compared to Cu‐PVP, the binding energy of the emission line at the N 1s orbital in CuZnO‐PVP shows a slight increase. This indicates a strong interaction between pyridine functional groups and ZnO, further supporting the aforementioned inference. Additionally, compared to other elements, the Zn 2p orbital XPS emission lines of the CuZnO‐PVP sample exhibit significantly lower intensity, indicating that the ZnO layer in the sample is extremely thin.

### 2.3 ECR Performance Characterizations

**Figure 3:**
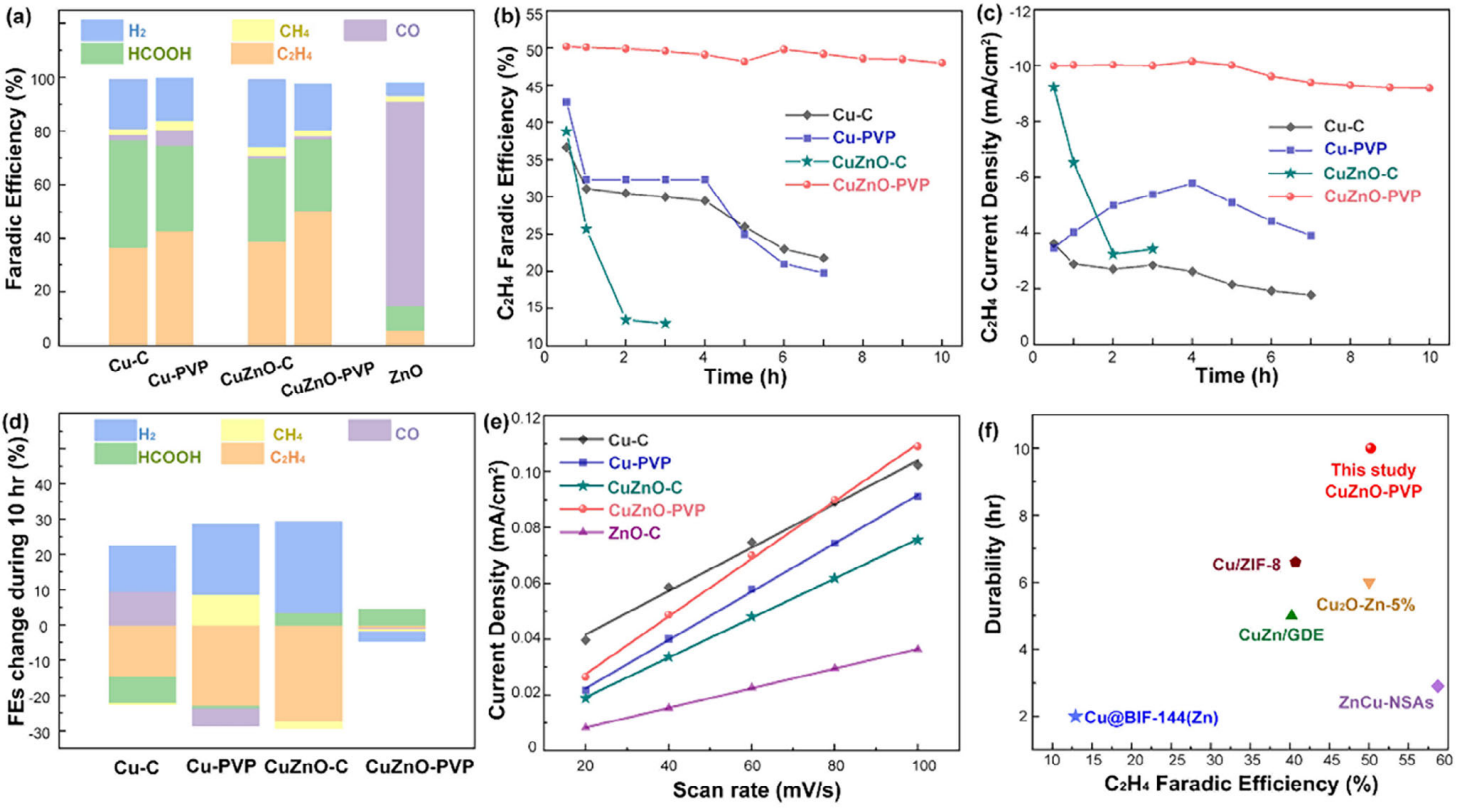
The ECR performance Cu‐C, Cu‐PVP, CuZnO‐C, and CuZnO‐PVP. a) Faradaic efficiencies (FEs) of the products, b) FEs and, c) current densities of C₂H₄ during the stability test, d) the changes in FE for each product during 10 h, e) Cdl results, and f) comparison between this work and others from literature.

**Figure** **3** presents the results of various characteristics during the ECR tests for the experimental samples (Cu─C, Cu‐PVP, CuZnO─C, and CuZnO‐PVP) compared to the control sample (ZnO─C). As shown in Figure 3a, the primary products are HCOOH and C_2_H_4_, with FE_C2H4_ and FE_HCOOH_ of 38% and 40%, respectively, while the FE_H2_ is 20%. This indicates that copper metal surfaces can simultaneously facilitate CO₂ and H₂O decomposition reactions. CO_2_ is adsorbed on the copper surface to form Cu‐CO_2_^ads^, while H atoms are generated from water. The interaction between Cu‐CO_2_^ads^ and H^+^ results in the formation of CO, hydrocarbons, and C_2_^+^. For the ZnO‐C sample, the primary ECR product is CO, consistent with most oxide‐based materials. This reflects the weaker E^ads^‐CO, leading to the rapid desorption of CO on ZnO surface.

Compared to Cu─C, the slight increase in FEs for C₂H₄, CH₄, and CO, accompanied by a decrease in HCOOH and H₂ efficiency, indicates that the PVP modification slightly enhances the formation of higher‐carbon products (C_2+_) on Cu NPs. It has been reported that PVP can regulate the valence states of Cu: when a sufficient amount of PVP is present, Cu remains in the Cu⁰ state, whereas PVP‐deficient Cu consists primarily of Cu₂O species, which predominantly produce CH₄ and C₂⁺ products, respectively.^[^ ^25^ ^]^ The interaction between PVP and metal surfaces can enhance CO₂ adsorption and suppress HER.^[^ ^24^, ^26^ ^]^ Besides, PVP can also promote the hydrophobic properties of the catalysts. The hydrophilic pyrrolidone end of the PVP molecule is bonded to the metal, while the hydrophobic carbon‐chain end remains exposed to the solvent. This interfacial bonding effect may alter the surface activity of the catalysts, thereby enhancing their catalytic performance.^[^ ^24^ ^]^ For CuZnO‐C, the FE_HCOOH_ decreases (9%), while FE_H2_ increases (≈7%), and FE_CO_ declines from 2% to 1.1%. As ZnO tends to produce CO, this result can be explained by the interfacial electronegativity coupling effect between the Zn and Cu NPs. The strong electronegativity difference localizes partial electrons from Zn atoms at the Cu‐ZnO interface and promotes CO adsorption, thus suppressing CO desorption. The increase in HCOOH and H₂ production suggests that residual metallic Zn atoms on the ZnO surface contribute to water decomposition. Compared to CuZnO─C, the surface modification with PVP further enhances the FE_C2H4_. The decrease in FE_H2_ indicates that functional groups reduce the number of active sites on the ZnO surface for water decomposition and increase those for C─C coupling. Consequently, the influence of PVP modification on selectivity can be attributed to two key factors: i) PVP ligands suppress HER, allowing more protons to participate in CO₂ reduction, and ii) PVP stabilizes *CO intermediates on Cu‐ZnO, promoting dimerization into C₂⁺ products. According to XPS analysis, this is likely due to the preferential adsorption of vinylpyrrolidone ligand on the ZnO surface. This hypothesis is further supported by subsequent in situ XAS analysis results.

During the stability test, all catalysts, except CuZnO‐PVP, exhibited significant changes in either product selectivity or current density. As shown in Figure 3b, the FE_C2H4_ dramatically decreased within the first hour, likely due to oxidation, aggregation, and morphological changes, as previously mentioned. In contrast, the FE_C2H4_ and the C_2_H_4_ current density of CuZnO‐PVP remained stable over 10 h, which can be attributed to the synergistic interaction between Cu nanoparticles, atomic‐layered ZnO, and PVP polymer layers. Furthermore, the decline in FE_C2H4_ during the stability test was accompanied by an increase in FE_H2_, as illustrated in Figure 3d.

To evaluate the electrochemical surface area (ECSA), the double‐layer capacitance (C_dl_) was measured. Figure 3e shows the slope of the current density differences against scan rates based on CV results obtained at scan rates ranging from 20 to 100 mV s^−1^. The CuZnO‐PVP catalyst exhibits the highest ECSA among all samples, as shown in Table S3 (Supporting Information), indicating the presence of abundant interfacial active sites. Compared to other Cu‐based catalysts, CuZnO‐PVP demonstrated superior stability and FE_C2H4_ performance, as highlighted in Figure 3f.

### 2.4 ECR Reaction Pathways Determination

**Figure 4:**
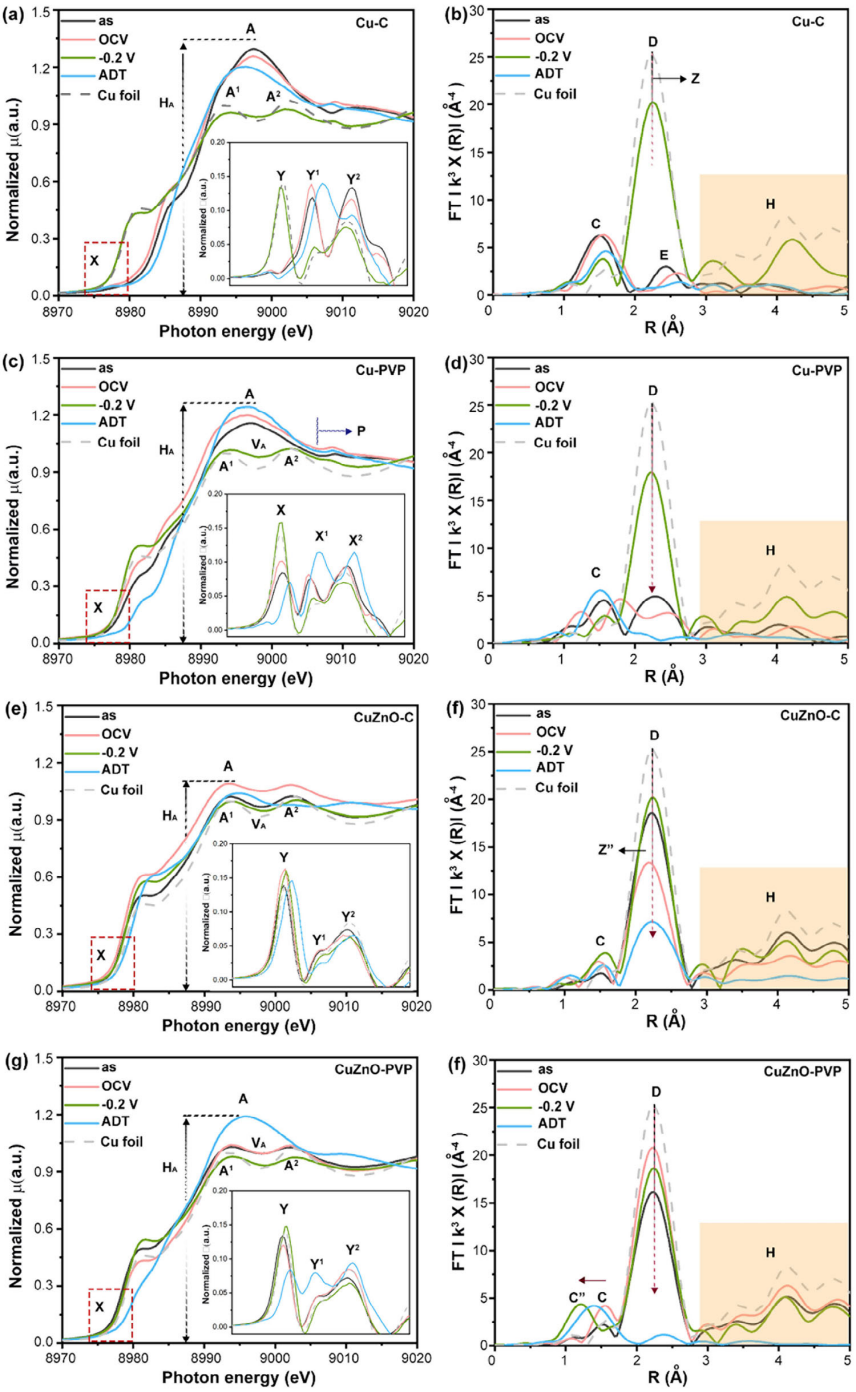
X‐ray absorption near‐edge structure (XANES) and extended X‐ray absorption fine structure (EXAFS) spectra of Cu‐C (a,b), Cu‐PVP (c,d), CuZnO‐C (e,f), and CuZnO‐PVP (g,h) under potential‐driven conditions of electrochemical CO_2_ reduction (ECR). Where as OCV, 0.2 V, and ADT denote samples at as‐prepared condition, open circuit potential, 0.2 V versus RHE, and after degradation test.

**Figure** **4**a presents XANES spectra of Cu‐C under various conditions: as‐prepared (AS), open circuit voltage (OCV), −0.2 V versus RHE, and post‐acceleration degradation test (ADT). Cu foil is included as a reference. Compared to the AS state, the spectra in region X shift to lower energy at OCV, indicating a slight reduction in copper oxidation. The corresponding changes in the Y^1^ and Y^2^ peaks of the first deviation curve also indicate the presence of adsorbates distinct from O^ads^ on the surface copper atoms (e.g., CO^ads^ in this study). At −0.2 V, Cu atoms are entirely reduced. After ADT, the Cu atoms are oxidized, thereby lowering C_2_H_4_ product efficiency in the long‐term ECR (Figure 3b). The Fourier‐transformed EXAFS spectra (i.e., radial structure function, RSF) of Cu─C are shown in Figure 4b, with the corresponding structural parameters summarized in Table S4 (Supporting Information). Accordingly, the radial peaks C, D, and E represent contributions from X‐ray interferences due to Cu─O, metallic Cu─Cu, and Cu─O (oxide), respectively. XANES profiles, RSF, and XRD confirm Cu‐C consists of a metallic Cu core (9.5–15 nm) with a Cu_2_O shell (5–8.6 nm). At OCV, Cu‐O (peak C) bond length increases, and Cu_2_O is consumed (peak E) via CO_2_ adsorption and decomposition. At −0.2 V, O atoms migrate to the surface (peak C), restoring metallic Cu (peak D), with minor residual oxygen (arrow Z).

Figure 4c,d show XANES and EXAFS spectra of Cu‐PVP. Cu NPs in Cu‐PVP exhibit mixed metallic and oxidized states in AS state. Although PVP can encapsulate Cu NPs, the polyvidone functional groups interact with Cu atoms primarily through steric effects via hydrogen bonding, consequently resulting in an inevitable thin oxidation layer on Cu. At OCV, the increase H_A_ (Figure 4c), a leftward shift in the near‐edge spectrum, and an increase in the intensity of pre‐edge peak X suggest the strong adsorption capacity of PVP's polyvidone ligands for CO₂ molecules. At −0.2 V, most Cu atoms remain in a metallic state (peaks A^1^ and A^2^ are similar to those of Cu foil). After ADT, Cu atoms are oxidized to form CuO, with partial metallic Cu retention (Figure 4c, inset).

Figure 4e,f illustrate XANES and EXAFS spectra of CuZnO─C. Cu remains predominantly metallic under all potentials, with ZnO forming a protective barrier against oxidation. At −0.2 V, Cu remains metallic with strong intermediate adsorption. ADT results in partial Cu oxidation (Figure 4e inset), confirming ZnO slows Cu oxidation, preserving core Cu metallicity but eventually leading to Cu‐Zn oxide formation, deactivating active sites for CO₂ reduction. As shown in Figure 4f, in the AS state, metallic Cu‐Cu/Cu‐Zn bonds (peak D) and Cu‐O^ads^ coordination (peak C) are present. Upon transitioning to OCV, Cu‐Cu/Cu‐Zn (peak D) coordination decreases, while Cu‐O^ads^ (peak C) coordination increases, indicating stronger O^ads^ binding and structural disorder. At −0.2 V, peak D intensity rises, restoring metallic Cu, while Cu─O coordination slightly increases. This suggests that the applied potential accelerates the ECR, reducing the duration of intermediate retention and restoring a substantial fraction of Cu atoms to a metallic state. Under ADT conditions, metallic Cu‐Cu (peak D) bonds are significantly suppressed, and high coordination shell signals (region H) are absent, indicating the coexistence of metallic Cu clusters and CuO. Figure 4g,h present CuZnO‐PVP XANES/EXAFS spectra. Cu remains metallic under all conditions except ADT, where oxidation occurs. However, the increased intensity at the V_A_ position suggests significant adsorption of O^ads^, leading to an increase in the average empty state of the 4s/4p orbitals. This phenomenon implies that PVP molecules preferentially interact with ZnO,^[^ ^33^ ^]^ exposing portions of the Cu NPs to air and resulting in the formation of Cu‐O^ads^. Additionally, the 1^st^ deviation peak (Y) is at a lower energy position compared to Cu foil, indicating that the 4s/4p energy band of Cu atoms is shifted to lower energies. At OCV, the near‐edge spectral shape remains unchanged while the intensity of the pre‐edge peak (X) decreases, indicating that Cu transitions to a more metallic state due to strong PVP/ZnO polarization effects. At −0.2 V, the intensity of the near‐edge region (A,^1^ A^2^ peaks, and V_A_) decreases significantly, indicating reduced vacancies in the 4s/4p orbitals (increased electron density) and a decrease in O^ads^ density. This further supports that PVP and ZnO structures weaken O^ads^ bonding, promoting its transfer to the Cu‐ZnO interface and regenerating active sites on the Cu NPs surface for H_2_O and CO_2_ splitting. This property allows Cu NPs to sustain more active sites for producing H^ads^. The increased intensity and blue shift of the pre‐edge peak (X) indicate an enhancement in Cu atom 3d orbital energy, increased electron density, and strengthened ligand field distortion, which complements the near‐edge spectral features. These characteristics suggest an enrichment of CO^ads^ on the surface of CuZnO‐PVP, significantly improving the yield and selectivity of multi‐carbon hydrocarbons. Furthermore, the weakened Cu─O^ads^ bonding by the PVP polyvidone ligand slows the oxidation of Cu, thereby prolonging the operational lifetime of CuZnO‐PVP in ECR. At ADT, the H_A_ intensity increases markedly, and the A^1^ and A^2^ peaks merge into a single peak (A), accompanied by a decrease in pre‐edge peak (X) intensity, and a blue shift, suggesting the oxidation of Cu. The first derivative curve of the spectrum (Figure 4g inset, and Figure S5, Supporting Information) reveals that the average chemical valence of Cu atoms lies between metallic and oxidized states. This feature is typical of metallic atom clusters embedded in oxides, where the interfacial stress between metal and oxide interfaces triggers exceptional activity at the reaction sites (e.g., Cu atoms in this study) in ECR. The EXAFS spectra(Figure 4h) and corresponding model analysis (Table S4, Supporting Information) further support the above scenarios. In AS state, the peaks C and D correspond to Cu─O and Cu─Cu/Cu─Zn bond contributions, with coordination numbers (CN) of 0.53 and 6.68/0.88, respectively. At OCV, the intensities of peaks C and D increase, reflecting higher CNs of Cu─O and Cu‐Cu bonds (1.49 and 8.84, respectively), while the CN of Zn remains unchanged. At −0.2 V (vs RHE), the CN_Cu‐Cu_ (peak D) decreases, while CN_Cu‐Zn_ drastically drops to 0.1, suggesting that the PVP polyvidone ligand accelerates Zn segregation from the Cu‐ZnO interface. In the ADT state, the CN_Cu‐Cu_ decreases to 0.38, while the ECR activity and selectivity remain stable, indicating that most metallic Cu atoms are distributed as small atomic clusters on the Cu NPs surface. Atomic‐scale Cu clusters and PVP‐ZnO interfaces sustain long‐term activity. Raman spectroscopy confirms that CuZnO‐C and CuZnO‐PVP initially contain Cu₂O and CuO, with the latter having less CuO (Figure S6, Supporting Information). During ADT, Cu₂O is reduced to metallic Cu under bias but later reoxidized to CuO.

Figure S7 (Supporting Information) presents wavelet‐transformed EXAFS (WT‐EXAFS) patterns for Cu‐C, Cu‐PVP, CuZnO‐C, and CuZnO‐PVP catalysts under ECR conditions. The radial distances (R‐axis) and peak widths (k‐axis) reflect bond lengths and electron cloud distributions, respectively, with broader peaks indicating weaker bonds. Peak intensities correlate with coordination numbers and structural order. The structural evolution of CuZnO‐PVP under ECR at −0.2 V and after ADT reveals unique advantages over Cu‐C, Cu‐PVP, and CuZnO‐C, linked to its stable CuZn active sites and synergistic atomic configuration. At −0.2 V, The WT‐EXAFS spectrum of CuZnO‐PVP shows a significant decrease in the metallic Cu M1 peak intensity and the near‐disappearance of the M2 peak. A distinct peak shift near the M2 region (R ≈ 1.3 Å) indicates the formation of CuZn hollow sites interacting with oxygen. These bimetallic sites are critical for stabilizing reaction intermediates (e.g., CO^ads^) and promoting C─C coupling for C₂H₄ production. In contrast, CuZnO‐C retains metallic Cu under the same bias but exhibits reduced interference intensity in coordination shells, suggesting weaker CO adsorption and fewer active sites. Besides, unlike Cu‐C and Cu‐PVP, which fully reduce to metallic Cu at −0.2 V (with short‐range order in Cu‐C and smaller nanoparticles in Cu‐PVP), CuZnO‐PVP's partial oxidation (due to PVP‐induced surface exposure) enables dynamic restructuring. The coexistence of CuZn sites and disordered Cu clusters allows complementary redox functionality, enhancing catalytic activity.

On the other hand, after ADT, CuZnO‐PVP retains only disordered Cu clusters (no higher‐shell interference peaks, R > 2.0 Å), and O adsorption signals (R ≈ 1.5 Å). Despite this structural disorder, C₂H₄ selectivity and current activity remain high (Figure 3), indicating that CuZn sites survive durability testing and drive sustained catalysis. In contrast, Cu─C reverts to stable Cu─O structures (peaks A″/F), losing metallic activity while Cu‐PVP oxidizes entirely (peak A), degrading performance. CuZnO‐C retains metallic Cu but forms a disordered surface oxide film, reducing C₂H₄‐active sites. The PVP enables Cu‐Zn interactions and ensures durability without sacrificing active sites, unlike CuZnO‐C, where ZnO alone suppresses oxidation but fails to prevent surface disorder post‐ADT.

**Scheme 1:**
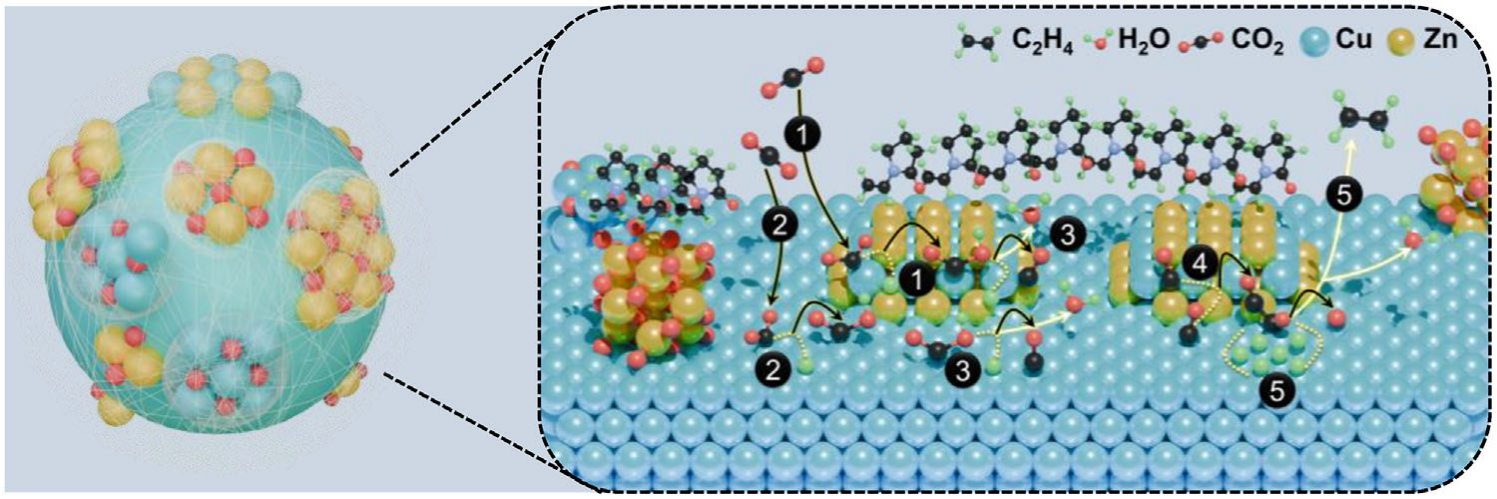
Schematic representation for the reaction pathways of ECR on CuZnO‐PVP surface according to the aforementioned Equation (1), (2), (3), (4), (5).

**Scheme** **1** illustrates the reaction pathway of CuZnO‐PVP during the ECR process, inferred by combining static physical and chemical structure analyses (STEM/EDS, XRD, XPS), in situ X‐ray absorption spectroscopy (Figure 3), electrochemical activity and durability tests (Figure 2), and in‐situ and ex situ Raman spectroscopy (Figures S3 and S6, Supporting Information) results. In the AS state, the CuZnO‐PVP comprises a metallic Cu nanocore surrounded by three coexisting nanoscale components: a CuZn nanoalloy in the near‐surface region, the metallic Cu nanoclusters with a surface layer of metastable oxide (Cu₂O), and a ZnO structure (a few atomic layers thick) partially covering the surface. The outermost layer is coated with a thin film of PVP molecule. Based on the CO_2_‐C_2_H_4_ conversion on Cu, the following mechanism can be proposed.^[^ ^25^ ^]^ At the OCV, Cu, and CuZn react with CO_2_ and form COOH^ads^ (Equations 1 and 2: CO_2_ adsorption). Besides, Cu and CuZn adsorb a significant amount of CO (CO^ads^) (Equation 3: initial reduction). When the applied voltage increases to −0.2 V versus RHE, CuZn and Cu enable coupling, forming a C─C bond (Equation 4) because abundant CuZn can increase asymmetric CO* binding and enhance C─C coupling.^[^ ^34^ ^]^ Subsequent protonation and reduction steps result in Cu‐O^ads^ and produce C_2_H_4_ (protonation and reduction). The CuZnO‐PVP shows high FE_C2H4_, surpassing most of the other Cu and CuZn‐based electrodes, as compared in **Table** **2**.

**Table 2:** The benchmark for the ECR performances of Cu‐Zn‐based electrocatalysts with similar metal and oxide components.

Samples	Electrolyte	V	FE_C2H4_	Stability	Refs.
		(vs. RHE)	(%)	(hr)	
CuZnO‐PVP/C	0.3 M KI +	−1	50.2	10	This work
	0.1 M KHCO_3_				
Cu@BIF‐144(Zn)	0.1 M KHCO_3_	−1.5	12.9	2	[18]
CuZn/GDE	2 M KCl +	−1.36	40.2	5	[16]
	0.01 M KHCO_3_				
Cu/ZIF‐8	1 M KOH	≈−1.65	40.7	6.6	[17]
ZnCu‐NSAs	0.5 M KHCO_3_	−1.14	58.7	‐	[35]
Zn2%‐OD Cu‐12h	0.1 M KHCO_3_	−1	≈30	‐	[19]
Cu_2_O‐Zn‐5%	1 M KOH	−1	≈50	6	[20]
Cu/GDL	2 M KCl	−1.14	41.7	30	[36]

CO_2_ adsorption:

(1) $$\mathrm{CuZn}+CO_{2}+H^{+}+e^{-}\to \mathrm{CuZn}-\mathrm{COO}H^{\mathrm{ads}}$$

(2) $$\mathrm{Cu}+CO_{2}+H^{+}+e^{-}\to \mathrm{Cu}-\mathrm{COO}H^{\mathrm{ads}}$$

Initial reduction:

(3) $$\begin{array}{ccc} \mathrm{CuZn}-\mathrm{COO}H^{\mathrm{ads}} & + & \mathrm{Cu}-\mathrm{COO}H^{\mathrm{ads}}+2H^{+}+2e^{-}\\ & & \to \mathrm{CuZn}-CO^{\mathrm{ads}}+\mathrm{Cu}-CO^{\mathrm{ads}}+2H_{2} \end{array}$$

C─C coupling:

(4) $$\mathrm{CuZn}-CO^{\mathrm{ads}}+\mathrm{Cu}-CO^{\mathrm{ads}}\to \mathrm{CuZn}-\mathrm{OCC}O^{\mathrm{ads}}-\mathrm{Cu}$$

Protonation and reduction:

(5) $$\begin{array}{ccc} \mathrm{CuZn}-\mathrm{OCC}O^{\mathrm{ads}}-\mathrm{Cu}+6H^{+}+6e^{-} & \to & \mathrm{Cu}-O^{\mathrm{ads}}+\mathrm{CuZn}\\ & + & C_{2}H_{4}+H_{2}O \end{array}$$

## 3 Conclusion

Metallic Cu NPs are widely recognized as one of the most effective materials for ECR to C_2+_, yet their practical application is hindered by rapid degradation, limited selectivity, and energy losses caused by metal corrosion, atomic reconstruction, particle aggregation, and surface oxidation. To address these challenges, we introduce a hierarchical nanocomposite composed of metallic Cu nanostructures capped with atomic ZnO layers and PVP polymer, synthesized through chemical adsorption and sequential reduction. The surface of this structure features CuZn dual‐atomic active sites that effectively adsorb CO^ads^​, and promote C─C coupling. The polyvidone ligands stabilize metallic Zn atoms on the ZnO surface and mitigate Cu oxidation. These synergistic mechanisms maintain a high electrochemically active surface area, even under partial Cu oxidation, resulting in a stable C_2_H_4_ selectivity of ≈50% and robust performance over 10 h of operation.

This work pioneers a triple‐phase heterointerface—organic (PVP), oxide (ZnO), and metal (Cu)—engineered at the atomic scale on Cu NPs. The hydrogen‐bond polarization effects of PVP ligands at the Cu and ZnO interfaces synergistically enhance both catalytic activity and durability. By achieving high‐current ECR for C_2_H_4_ production while extending catalyst lifespan, this design offers a transformative strategy for CO_2_ utilization. The concept of sub‐nanometer organic‐metal‐oxide interfaces holds broad promise, potentially inspiring advancements in diverse electrocatalytic systems.

## Conflict of Interest

The authors declare no conflict of interest.

## Supporting information



Supporting Information

## Data Availability

The data that support the findings of this study are available from the corresponding author upon reasonable request.
