# Supplementary material for: Atomic Layered ZnO Between Cu Nanoparticles and a PVP Polymer Layer Enable Exceptional Selectivity and Stability in Electrocatalytic CO2 Reduction to C2H4
Source: Adv Sci (Weinh). 2025 Apr 26;12(26):2501642. doi: 10.1002/advs.202501642 (PMC12244499; doi:10.1002/advs.202501642)
Supplement: Supplementary file 1 — Supporting Information [file ADVS-12-2501642-s001.docx]

**Supplementary information for**

**Atomic Layered ZnO between Cu Nanoparticles and a PVP polymer layer Enable Exceptional Selectivity and Stability in Electrocatalytic CO_2_ Reduction to C_2_H_4_**

Lihui Zhou ^a,o^, Hung-Wei Tsai ^b,o^, Ting-Wei Kuo ^b^, Jui-Cheng Kao ^c^, Yu-Chieh Lo ^c^, Ji-Min Chang ^d^, Tzu-Hsuan Chiang ^d^, Sheng Dai ^a*^, Kuan-Wen Wang ^b*^ and Tsan-Yao Chen ^e,f,g*^

^a.^ Key Laboratory for Advanced Materials and Feringa Nobel Prize Scientist Joint Research Centre, School of Chemistry and Molecular Engineering, East China University of Science & Technology, Shanghai 200237, China

^b.^ Institute of Materials Science and Engineering, National Central University, Taoyuan City 32001, Taiwan

^c.^ Department of Materials Science and Engineering, National Yang Ming Chiao Tung University, Hsinchu 30010, Taiwan

^d.^ Department of Energy Engineering, National United University, Miaoli 360301, Taiwan.

^e.^ Department of Engineering and System Science, National Tsing Hua University, Hsinchu 30013, Taiwan

^f.^ Institute of Analytical and Environmental Science, National Tsing Hua University, Hsinchu 30013, Taiwan

^g.^ Institute of Nuclear Engineering and Science, National Tsing Hua University, Hsinchu 30013, Taiwan

*Corresponding Author:

Kaun-Wen Wang

Institute of Materials Science and Engineering,

National Central University, Taoyuan City 32001, Taiwan

Email: [Kuanwen.Wang@gmail.com](mailto:Kuanwen.Wang@gmail.com)

Sheng Dai

Key Laboratory for Advanced Materials and Feringa Nobel Prize Scientist Joint Research Centre,

School of Chemistry and Molecular Engineering,

East China University of Science & Technology, Shanghai 200237, China

Email: [shengdai@ecust.edu.cn](mailto:shengdai@ecust.edu.cn)

Prof. Tsan-Yao Chen

Department of Engineering and System Science,

National Tsing-Hua University, Hsinchu 300, Taiwan

Email: chencaeser@gmail.com / FAX: +886-3-5720724

**Preparation of Catalysts**

**1.** Materials

All reagents in this work were used as received without further purification. Copper (II) acetate (Cu(CH_3_COO)_2_, 99.999%) and oleylamine (OAm, 80-90%) were purchased from Thermo Fisher Scientific. Oleic acid was purchased from SHOWA Chemicals Inc. Potassium hydrogen carbonate (KHCO_3_, 99%) was purchased from Fisher Chemical. Zinc acetate (Zn(CH_3_COO)_2_, 99.99%) was manufactured by Sigma-Aldrich. Vulcan^®^ XC-72 was purchased from Cabot Corporation. Polyvinylpyrrolidone (PVP) was purchased from Emperor Chemical Co., Ltd. Deionized (DI) water was prepared with an ultra-pure purification system in which the resistivity was controlled at 18.2 MΩ cm^-1^.

2. Synthesis of CuZnO-C catalysts

Carbon-supported CuZnO nanoparticles (NPs) with metal loading of 30 wt% and Cu/Zn atomic ratio of 93/7 were synthesized by the OAm method. First, Zn(CH_3_COO)_2_ was mixed with 9 ml of OAm and 1 ml of oleic acid in a three-neck flask, heated to 343K for 10 minutes under N_2_, and then heated to 543K for 1 hour. Next, Cu(CH_3_COO)_2_ and 3 ml of OAm were added into the above solution at 543K for 1 hour. Finally, XC-72 powder was mixed in the solution and stirred for 24 hours. The resulting products were centrifuged, rinsed with ethanol/hexane mixture, and dried for 1 day to obtain CuZnO-C. For comparison, Cu-C and ZnO-C were synthesized using the same OAm method without using ZnO precursors and Cu precursors, respectively.

3. Synthesis of CuZnO-PVP catalysts

The as-prepared samples were surface-modified by PVP. 6 mg of as-prepared samples was added to the PVP suspensions (0.24 g PVP in 6.25 ml DI water), and stirred at room temperature for 12 h. Subsequently, the catalysts were collected by centrifugation and dried for 1 day to obtain CuZnO-PVP.

4. Characterizations of Catalysts

The X-ray diffraction (XRD) patterns were obtained using a Bruker D8 Advance diffractometer with Cu Kα radiation (λ = 1.54 Å, 40 kV, and 40 mA) to examine the crystal structure and average grain size of the catalysts. The measurements were performed in the 2θ range of 20-80° at a scan rate of 0.15° per step. The inductively coupled plasma-optical emission spectrometer (ICP-OES) analysis was performed using an Agilent 725 to determine the exact metal loading of the catalysts. X-ray photoelectron spectroscopy (XPS) studies were conducted using a Thermo VG- Scientific Sigma Probe equipped with a monochromatic Al Kα X-ray source (hν = 1486.6 eV) at a voltage of 20 kV and a current of 30 mA. All binding energies were calibrated using the C 1s peak at 284.6 eV.

The ex-situ X-ray absorption spectroscopy (XAS) analysis, which includes X-ray absorption near-edge structure (XANES) and extended X-ray absorption fine structure (EXAFS), was conducted. Both ex-situ and in-situ XAS spectra were obtained from the TLS-17C1 and TPS-44A1 beamlines at the National Synchrotron Radiation Research Center (NSRRC, Taiwan) for the Cu K-edge (8979 eV) and Zn K-edge (9659 eV), respectively. For the in-situ measurements, the prepared catalysts were drop-cast onto a carbon plate. A platinum wire served as the counter electrode, and Ag/AgCl (3M KCl) as the reference electrode in a solution of 0.1 M KHCO_3_ with 0.3 M KI.

A customized electrochemical cell made of polytetrafluoroethylene (PTFE) with a rectangular hole (2 × 2 cm^2^) was used for in-situ XAS experiments. The XAS spectra were collected at the Cu K-edge of the samples under various conditions: as-prepared, at open circuit voltage (OCV), at -0.2 V versus RHE, and after the durability test.

CO stripping tests were conducted using a potentiostat (CHI 612E) in a three-electrode cell. CO adsorption on the catalyst surface was achieved by purging CO into 0.5 M H_2_SO_4_ at 0.05 V for 30 minutes. Subsequently, CO stripping voltammetry was performed in an N_2_-saturated 0.5 M H_2_SO_4_ solution, scanning from -0.10 to 1.20 V at a rate of 50 mV s⁻¹. The first cycle recorded the CO^ads^ stripping, while the second cycle confirmed the complete removal of CO^ads^ during the initial scan.

In-situ Raman spectroscopy was performed using a Reflex Raman microscope (UniDRON) with a 473 nm HeNe laser, and a Leica N Plan 50×/0.75 BD objective lens was utilized to monitor the real-time evolution of the surface states of electrocatalysts under different applied voltages between 0 to -1.6V vs. RHE. The electrochemical measurements were used in a Teflon cell configured in a standard three-electrode setup, where the working electrode consisted of various catalysts loaded onto nickel foam. A platinum sheet served as the counter electrode, and an Ag/AgCl electrode was used as the reference electrode. All experiments were carried out in a CO_2_-saturated 0.1 M KHCO_3_ electrolyte.

5. CO_2_RR Measurement of Catalysts

The CO_2_RR performance of the catalysts was evaluated in a gas-tight H-cell, which included a two-compartment separated by a cation exchange membrane (Nafion 212). The electrochemical results of catalysts were conducted using CH Instruments 612E. A carbon rod and Ag/AgCl (3M KCl) were used as counter and reference electrodes, respectively. The electrolyte (0.3M KI, with a small amount of 0.1 M KHCO_3_ as a buffer solution) was continuously introduced into both compartments, while ultrahigh purity carbon dioxide (99.9995 %) was continuously supplied at a flow rate of 40 sccm (mL min^-1^) for over 30 minutes. All potentials recorded using Ag/AgCl were converted to a reversible hydrogen electrode (RHE) according to the following equation:

| $E_{RHE}=E_{\left( vs.\frac{Ag}{AgCl} \right)}+0.210V+ 0.0591 \times pH$ |  | (eq. 1) |
| --- | --- | --- |

The catalyst ink was prepared by ultrasonically dispersing 1 mg of the sample powder with 10μL of Nafion solution (5 wt %) and 200 μL of IPA for 30 min. 13.4 μL of the catalyst ink was drop-coated on the glassy carbon electrode (GCE, 0.2827 cm^2^).

The gaseous products were detected using a gas chromatograph (GC, Agilent 6890N) equipped with a pulsed discharge helium ionization detector (PDHID, model D-3-I-7890, VICI, USA) and a carbon molecular sieve-packed column (Shincarbon ST, 2 m x 1.0 mm i.d.; Restek Chromatography Products, USA) for the quantification of H_2_, CO, CH_4_, and C_2_H_4_. Ultrahigh purity helium (99.999%) was used as the carrier gas. Calibration curves were generated using five different concentrations ranging from 50 to 150 ppm to calibrate the volume ratio of each product.

| ${FE}_{gas}(\%)=\frac{\alpha nF}{Q}=\frac{\alpha\times ppm\times C_{CO2}\times t\times F}{Q}\times100\%$ | (eq. 2.2) |
| --- | --- |
|  |  |

The Faradaic efficiency of gas was determined by the following formula [1]:

, here α represents the number of transferred electrons for the production of H_2_, CO, CH_4_, and C_2_H_4_, ppm denotes the concentration of each gas product, C_CO2_ indicates the flow rate of CO_2_ (40 mL/min), t stands for the reaction time, F represents Faraday's constant (96485 C/mol), and Q (A·s) represents the total electric charge quantity.

To determine the double-layer capacitance (C_dl_) and electrochemical active area (ECSA) of the samples employed in CO_2_RR, cyclic voltammetry (CV) tests were conducted at different scan rates using a CH Instruments 6116E in a 3-electrode system. Each sample underwent CV testing in a 0.3 M KI electrolyte with scan rates ranging from 20 to 100 mV/s. The scan range was chosen to include the region where no significant redox reactions occurred in both positive and negative scans, typically with 0.1 V. The difference in average current density (ΔJ) between cathodic (J_C_) and anodic (J_A_) current densities in this selected range was calculated to determine the slope of ΔJ versus scan rates, which corresponds to the double-layer capacitance (C_dl_) of each sample as per equations 3 and 4 [2].

$\Delta J\left( \frac{\mathrm{mA}}{{cm}^{2}} \right)=\frac{J_{A}-J_{C}}{2} (\frac{mA}{{cm}^{2}})$ (eq. 2.3)

$ECSA \left( {cm}^{2} \right)=\frac{C_{dl} (mF{cm}^{-2})}{C_{s} (mF{cm}^{-2})}\times S ({cm}^{2})$ (eq. 2.4)
, where C_dl_ denotes the double-layer capacitance derived from the CV curves. C_s_, the assumed specific capacitance, is fixed at 0.029 mF/cm², representing the typical double-layer capacitance of a smooth metal surface, and S denotes the actual geometric surface area of the electrode.

1. X-ray Diffraction analysis on Nanocomposites of Cu-supported ZnO with PVP encapsulation


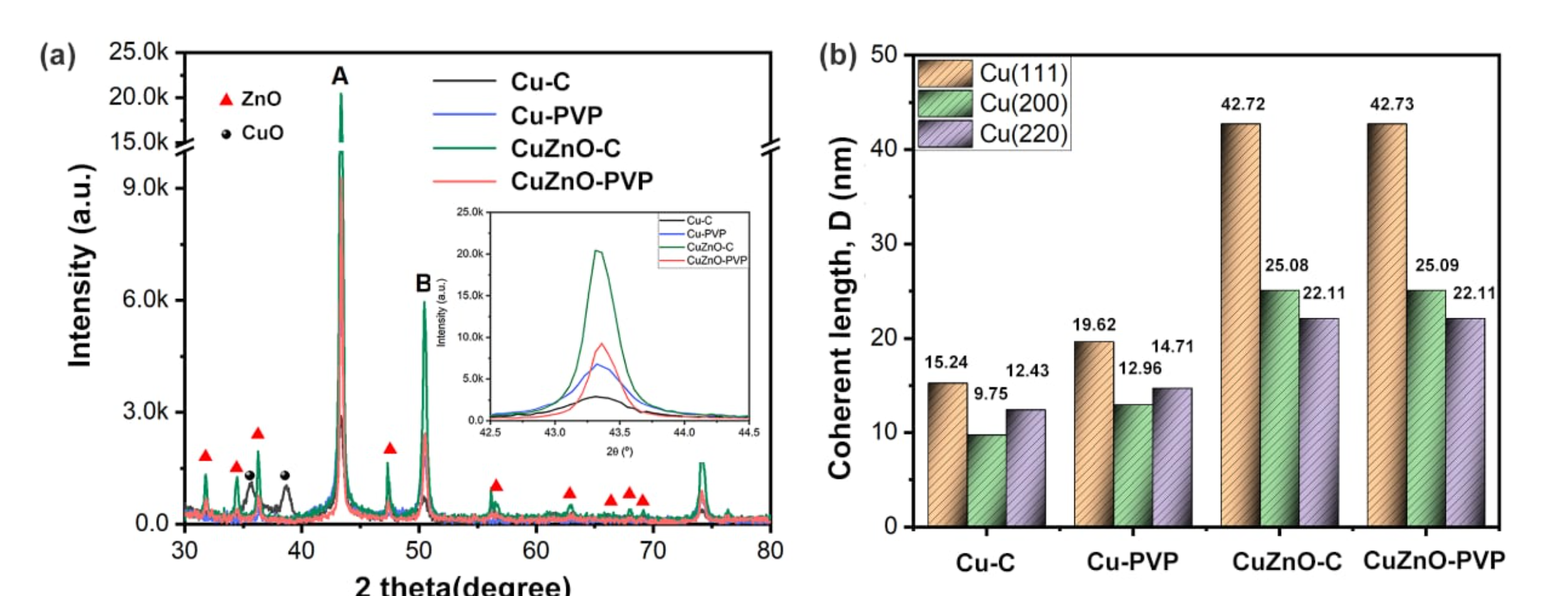


**Figure S1** (a) XRD patterns of Cu-C, Cu-PVP, CuZnO-C and CuZnO-PVP and (b) corresponding coherent lengths of (111), (200) and (220) facets for metallic Cu region.

**Table S1** XRD determined the structure parameters of experimental samples.

|  | d spacing (Å) | | | | | D (nm) | | | | |
| --- | --- | --- | --- | --- | --- | --- | --- | --- | --- | --- |
| sample | Cu (111) | Cu (200) | Cu (220) | CuO (1) | CuO (2) | Cu (111) | Cu (200) | Cu (220) | CuO (1) | CuO (2) |
| Cu-C | 2.087 | 1.809 | 1.280 | 2.513 | 2.324 | 155.351 | 97.523 | 124.273 | 55.620 | 84.169 |
| Cu-PVP | 2.082 | 1.802 | 1.276 | NA | NA | 196.233 | 129.632 | 147.036 | NA | NA |

|  | d spacing (Å) | | | | | | D (nm) | | | | | |
| --- | --- | --- | --- | --- | --- | --- | --- | --- | --- | --- | --- | --- |
|  | Cu (111) | Cu (200) | Cu (220) | ZnO (1) | ZnO (2) | ZnO (3) | Cu (111) | Cu (200) | Cu (220) | ZnO (1) | ZnO (2) | ZnO (3) |
| CuZnO-C | 2.087 | 1.807 | 1.278 | 2.811 | 2.601 | 2.476 | 427.218 | 250.824 | 221.148 | 330.291 | 277.144 | 334.242 |
| CuZnO-PVP | 2.085 | 1.805 | 1.278 | 2.807 | 2.601 | 2.472 | 427.292 | 250.876 | 221.148 | 235.951 | 97.815 | 278.575 |

1. **
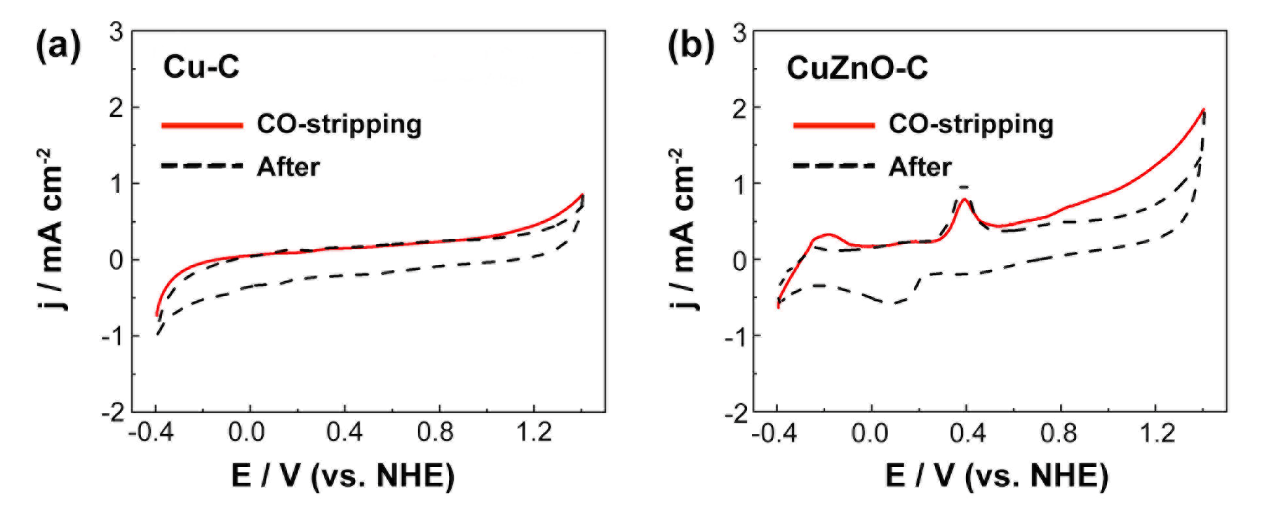
**CO-stripping data of Cu-C and CuZnO-C.

**Figure S2** The CO-stripping data of Cu-C and CuZnO-C.


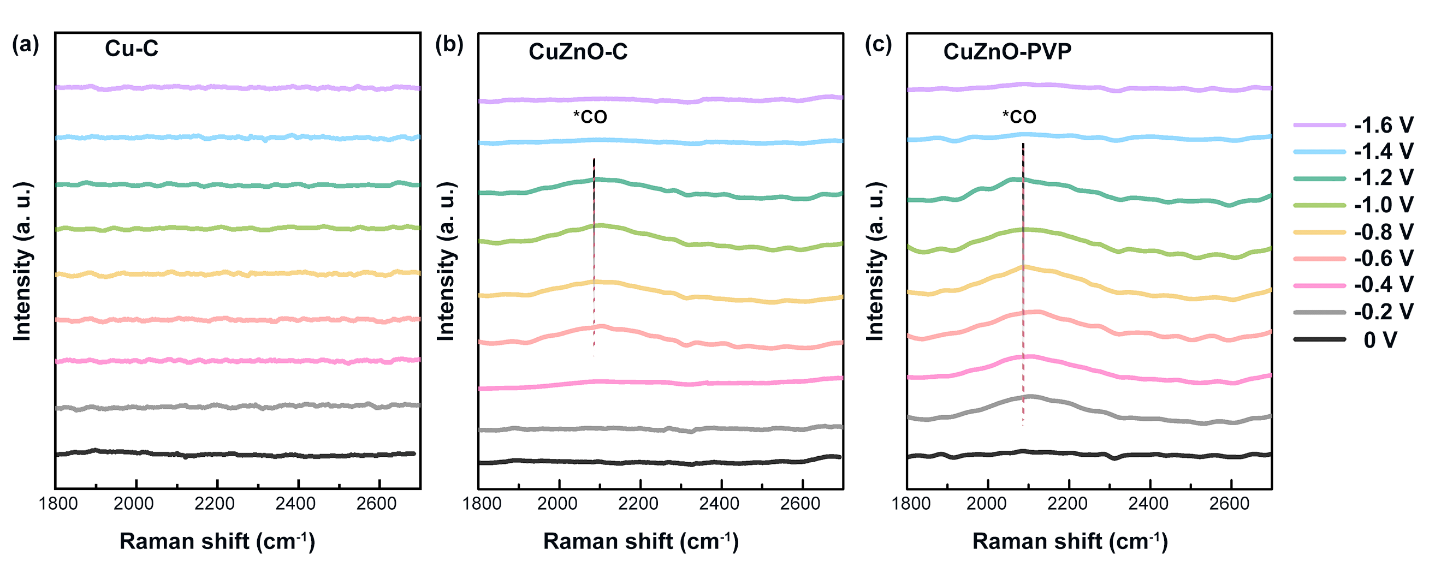
3. In situ Raman spectra

**Figure S3** In situ Raman spectra of (a) Cu-C, (b) CuZnO-C, and (c) CuZnO-PVP

obtained in a potential window 0 to −1.6 V.

4. XPS analysis on Cu-C, CuZnO-C, Cu-PVP, and CuZnO-PVP.

**Figure S4** XPS spectra (symbols) and fitted curves (red solid lines) for Cu-C, Cu-PVP, CuZnO-C, and CuZnO-PVP at the Cu 2p, Zn 2p, and N 1s orbitals. In the Cu 2p spectra, the green and blue lines represent the deconvoluted curves of Cu⁰ and Cu²⁺, respectively.

**Table S2** XPS determined surface binding energy and composition experimental samples.

|  | Surface composition | | | | |
| --- | --- | --- | --- | --- | --- |
| Sample | Cu/Zn | Cu^2+^ | Cu^0^ | Zn^2+^ | Zn^0^ |
| Cu-C | 100 | 25 | 75 |  |  |
| Cu-PVP | 100 | 21 | 79 |  |  |
| CuZnO-C | 68/32 | 21 | 79 | 100 | 0 |
| CuZnO-PVP | 71/29 | 49 | 51 | 100 | 0 |
| ZnO-C | 0/100 |  |  | 100 | 0 |

5. C_dl_ and ECSA data of samples.

**Table S3** C_dl_ and ECSA results of samples in 0.1M KHCO_3_ with 0.3 M KI electrolyte.

| **Samples** | **C_dl_ (mF/cm^2^)** | **ECSA (cm^2^)** |
| --- | --- | --- |
| Cu-C | 0.8 | 27.6 |
| Cu-PVP | 0.9 | 31.0 |
| CuZnO-C | 0.7 | 24.1 |
| CuZnO-PVP | 1.0 | 34.5 |
| ZnO-C | 0.4 | 13.8 |

6. X-ray absorption spectroscopy analysis on CuZnO-PVP


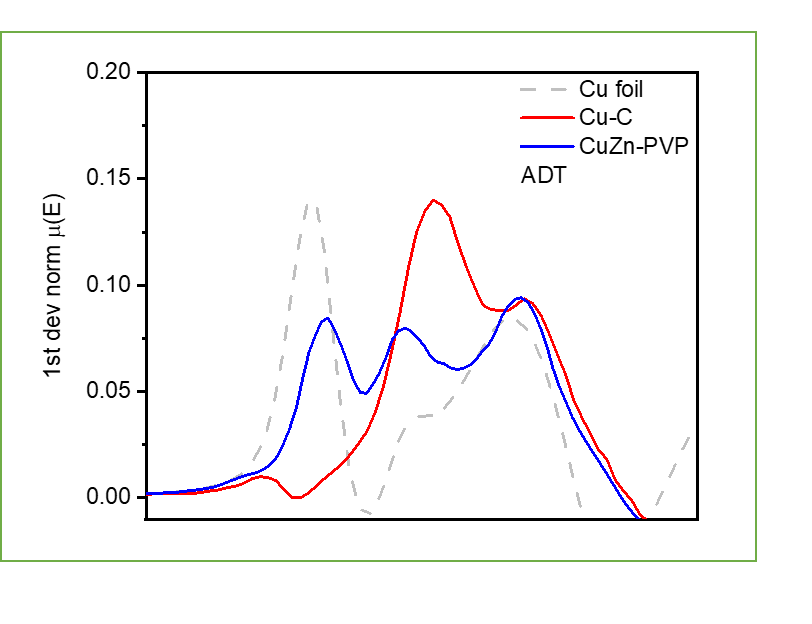


**Figure S5** Enlarged 1^st^ deviation curves of XAS spectra in **Figure 4(g)** inset.

**Table S4** XAS fitting results of Cu-C, Cu-PVP, CuZnO-C and CuZnO-PVP at Cu K-edge in potential driven conditions of ECR.

| Cu-C | CN | | | R (Å) | | |
| --- | --- | --- | --- | --- | --- | --- |
|  | Cu-O^ads^ | Cu-O | Cu-Cu^M^ | Cu-O^ads^ | Cu-O | Cu-Cu^M^ |
| as | 4.35 | 3.35* |  | 1.947 | 2.997* |  |
| OCV | 4.11 | 1.45* |  | 1.971 | 2.941* |  |
| -0.2V | 1.31 |  | 9.59 | 1.969 |  | 2.547 |
| ADT | 3.01 |  | 0.35 | 1.998 |  | 2.729 |

| Cu-PVP | CN | | | R (Å) | | |
| --- | --- | --- | --- | --- | --- | --- |
|  | Cu-O^ads^ | Cu_2_O | Cu-Cu^M^ | Cu-O^ads^ | Cu_2_O | Cu-Cu^M^ |
| as | 2.65 |  | 2.65 | 1.983 |  | 2.592 |
| OCV | 1.96 | 1.36 | 2.93 | 1.883 | 2.639 | 2.496 |
| -0.2V |  |  | 8.71 |  |  | 2.529 |
| ADT | 4.07 |  |  | 1.979 |  |  |

| CuZnO-C | CN | | | | R (Å) | | | |
| --- | --- | --- | --- | --- | --- | --- | --- | --- |
|  | Cu-O^ads^ | Cu-CO^ads^ | Cu-Cu^M^ | Cu-Zn | Cu-O^ads^ | Cu-CO^ads^ | Cu-Cu^M^ | Cu-Zn |
| as | 0.34 |  | 8.01 | 0.89 | 1.846 |  | 2.533 | 2.533 |
| OCV | 0.71 |  | 5.51 | 0.87 | 1.837 |  | 2.517 | 2.517 |
| -0.2V | 1.45 | 1.13 | 8.36 | 0.58 | 1.869 | 2.534 | 2.537 | 2.537 |
| ADT | 0.94 | 0.68 | 3.13 | 0.58 | 1.874 | 2.786 | 2.547 | 2.547 |

| CnZnO- PVP | CN | | | | | R (Å) | | | | |
| --- | --- | --- | --- | --- | --- | --- | --- | --- | --- | --- |
|  | Cu-O^ads^ | Cu-H_2_O^ads^ | Cu-CO^ads^ | Cu-Cu^M^ | Cu-Zn | Cu-O^ads^ | Cu-H_2_O^ads^ | Cu-CO^ads^ | Cu-Cu^M^ | Cu-Zn |
| as | 0.53 |  |  | 6.68 | 0.88 | 1.877 |  |  | 2.533 | 2.533 |
| OCV | 1.49 |  |  | 8.84 | 0.88 | 1.907 |  |  | 2.518 | 2.518 |
| -0.2V | 2.89 | 0.5 | 8.72 | 8.02 | 0.1 | 1.731 | 2.027 | 2.829 | 2.504 | 2.504 |
| ADT | 3.11 |  | 1.05 | 0.38 | 0.1 | 1.914 |  | 2.821 | 2.523 |  |

* denotes the bond pairs of Cu_2_O

7. Raman spectroscopy analysis on Cu-C, Cu-PVP, CuZnO-C and CuZnO-PVP at as-prepared and post-ADT conditions.

**

**

**Figure S6** Raman spectra of as-prepared and post-ADT samples.

8. In-situ X-ray absorption analysis on Cu-C, Cu-PVP, CuZnO-C and CuZnO-PVP at potential driven conditions of ECR.

**Figure S7** illustrates the Wavelet-Transformed (WT) EXAFS patterns for Cu-C, Cu-PVP, CuZnO-C, and CuZnO-PVP under potential-driven conditions during the electrochemical CO₂ reduction reaction (ECR). Taking peak A as an example, the contour peak's radial distance (RA) along the R-axis from the origin represents the bond length between Cu and the surrounding A atoms. The scattering peak's width along the k-axis (∆kA, B...M) corresponds to the spatial probability distribution function of the electron cloud associated with the A-Cu bond. A broader distribution indicates greater kinetic energy dispersion of electrons between the two atoms, signifying a weaker bond between A and Cu. The peak intensity is proportional to the coordination number and the degree of local structural order. For reference and discussion, the WT-EXAFS pattern of standard metallic copper is shown in Figure S6. In this standard, scattering peaks in regions I (2.3–3.0 Å) and II (3.5–5.4 Å) represent X-ray interference contributions from Cu atoms in the first and second coordination shells, respectively.

Based on the aforementioned description, the copper atoms in the as-prepared Cu-C state coexist in a mixed state, consisting of a small amount of metallic Cu (peak B) and two distinct oxides. Peaks A and C originate from Cu₂O, while peak D is attributed to contributions from distant O atoms around Cu, associated with an amorphous Cu oxide (CuOₐₘₒᵣ) structure containing numerous defects. Under open-circuit voltage (OCV) conditions, the intensity of peak D nearly disappears, and peaks C and B merge into a single scattering peak (B"). The position of peak A remains unchanged, but its intensity increases. The evolution of peaks B, C, and D indicates the gradual reduction of oxides to partial metallic states and the consumption of CuOₐₘₒᵣ under applied bias and interaction with CO₂ molecules. The enhanced intensity of peak A is attributed to the adsorption of CO, generated from CO₂ dissociation, on the Cu surface. At high bias (0.2 V vs. RHE), all copper oxides are fully reduced, transforming all Cu atoms into the metallic state. Peaks M^1^ and M^2^ correspond to X-ray interference signals from Cu atoms in the first and second coordination shells within the metallic Cu lattice, with respective bond distances of R_Cu-CuM1_ and R_Cu-CuM2_. Compared to the structure of metallic copper foil (**Figure S8**), only high wavevector interference (k = 11.5 Å⁻¹) is observed for peak M2, which is characteristic of the short-range ordered nanoscale metallic Cu structure. After accelerated durability testing (ADT), the metallic Cu features almost completely disappear, with most atoms reverting to local Cu oxide structures, as indicated by peaks A" and F. The significantly broad spatial distribution of peak B (2.5–5.0 Å) suggests the formation of a stable oxide structure with strong Cu-O bonds and a variety of Cu-O bond lengths within the atomic structure.

In the as-prepared state, the Cu-PVP sample exhibits a coexistence of metallic Cu (M^1^) and oxide (F) states. The contour peak near 5.1 Å from the central atom corresponds to contributions from oxygen atoms within the PVP. Under open-circuit voltage (OCV) conditions, the metallic Cu signal nearly vanishes, which is attributed to the significant adsorption of CO on surface Cu atoms. Since the Cu atoms are uniformly distributed on the activated carbon support with only a few nanometers in thickness, the extensive surface chemisorption of CO under these conditions results in a transformation of most Cu atoms into a quasi-oxide-like structure due to CO adsorption. At 0.2 V vs. RHE, the WT-EXAFS spectrum of Cu atoms resembles that of Cu-C. A slight decrease in the peak intensity of the interference signals indicates a smaller dimension of the metallic Cu structure, which aligns with the observations from the STEM/EDS images (**Figure 1**). In the ADT state, most of the metallic Cu structure converts to an oxidized form, as evidenced by peak A. Peaks D and F" represent contributions from a stable Cu oxide structure.

In the as-prepared state, the Cu atomic structure in CuZnO-C exhibits metallic characteristics. Compared to Cu-PVP, its peak D is split into two interference peaks similar to those of the metallic phase. This indicates that the atomic ZnO thin layer effectively suppresses local structural disorder in Cu atoms caused by surface oxidation. Under open-circuit voltage (OCV) conditions, the interference intensity in the M1 and M2 regions decreases, similar to the behavior observed in the previously discussed samples. At 0.2 V vs. RHE, the interference intensity of the two coordination shells further diminishes. Additionally, the left-side interference peak in the M1 region shows significantly reduced intensity, suggesting that more CO^ads^ is adsorbed on some surface Cu atoms. In the ADT state, the Cu atoms largely retain their metallic state. However, the broad interference peak in the M2 region indicates the formation of a disordered oxide film on the sample surface. This disorder reduces the number of active sites on CuZnO-C available for the production of C₂H₄.

Compared to CuZnO-C, the metallic structure of CuZnO-PVP in the as-prepared state exhibits lower order. This suggests that PVP molecules tend to encapsulate the ZnO surface, leaving the Cu surface exposed to the environment, resulting in partial oxidation. Under open-circuit voltage (OCV) conditions, the interference peak intensity slightly decreases, consistent with observations in other materials. At 0.2 V vs. RHE, the interference intensity of the metallic Cu M^1^ peak decreases significantly, while the M^2^ peak nearly disappears. Additionally, the peak to the right of M^2^ shifts closer to the central atom, indicating the formation of Cu₂Zn sites on the surface, involving a certain proportion of Cu and Zn atoms. The contour peak near 1.3 Å corresponds to interference signals from Cu₂Zn hollow sites interacting with oxygen (O). In the ADT state, only interference peaks corresponding to O^ads^ (R ≈ 1.5 Å) can be observed around the Cu atoms. In the higher-order shell region (R > 2.0 Å), there is no detectable interference intensity, a characteristic of disordered Cu atomic clusters. Comparing this structural evolution to the ECR performance results (**Figure 2**), the material retains high current activity and C₂H₄ selectivity. This suggests that the Cu₂Zn sites remain intact, while the surrounding atoms continue to provide complementary redox functionality for the reaction.


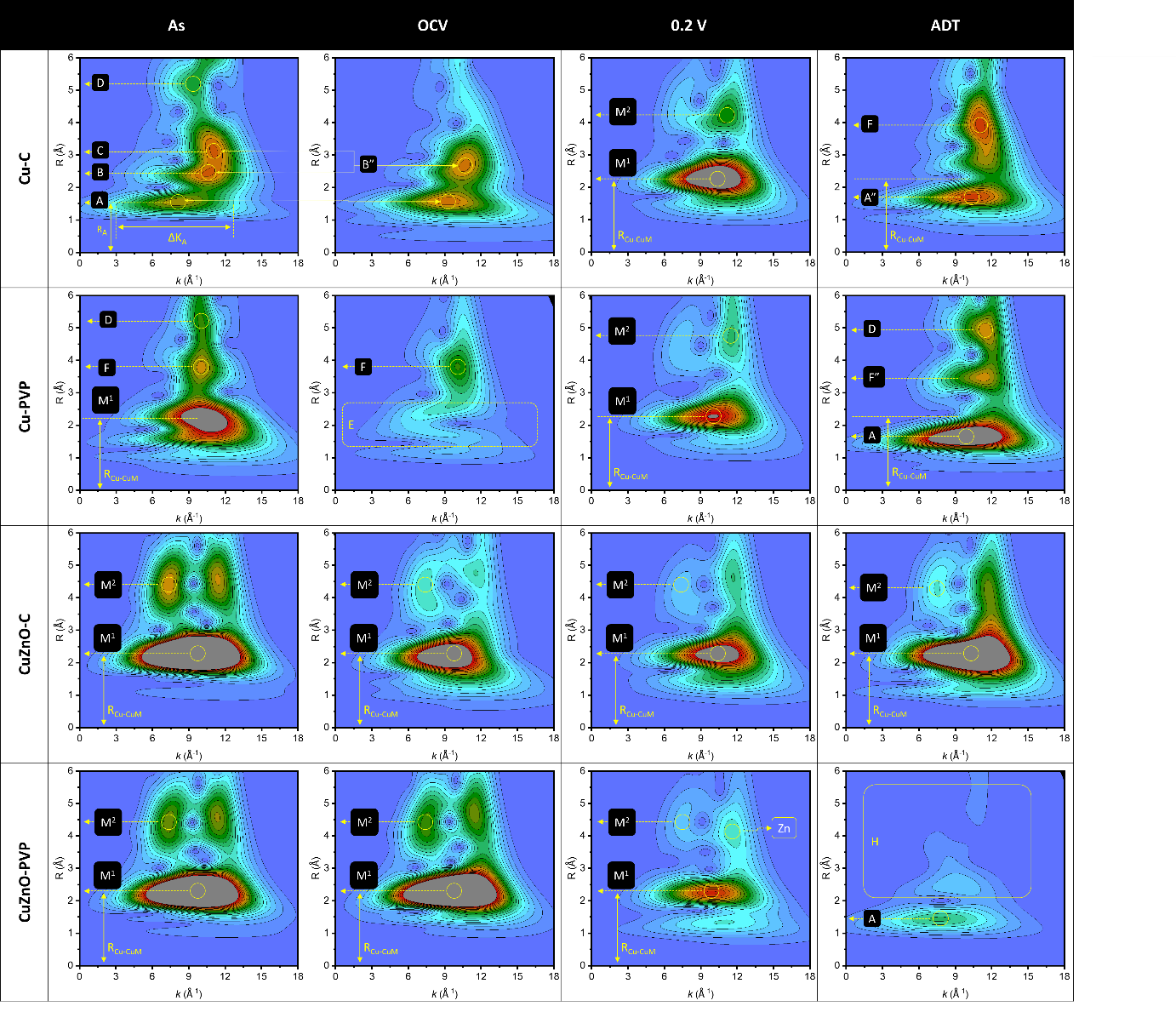


**Figure S7** Wavelet-Transformed EXAFS patterns of Cu-C, Cu-PVP, CuZnO-C and CuZnO-PVP at potential driven conditions of ECR.


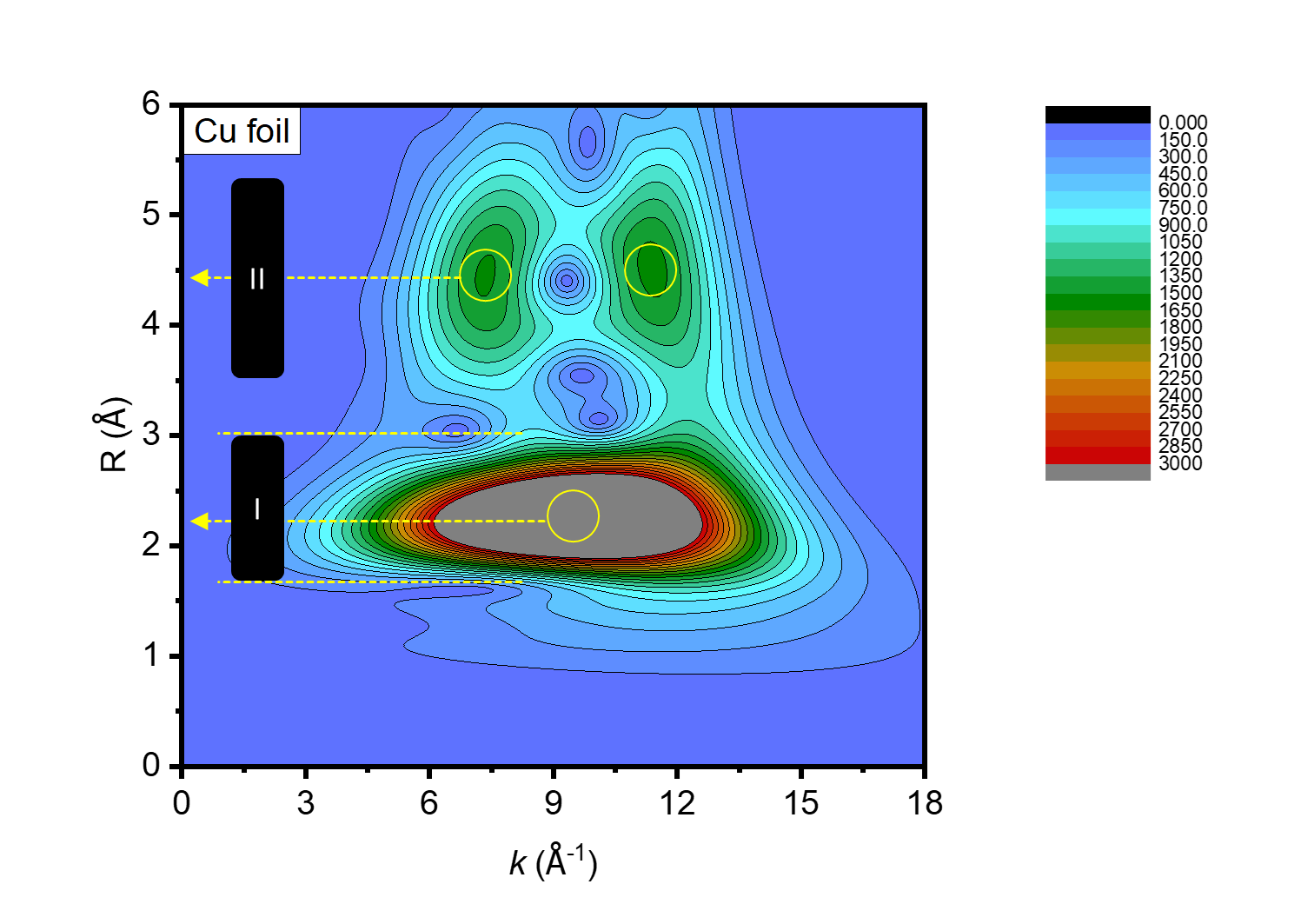


**Figure S8**. WT-EXAFS pattern of Cu foil.

**References**

[1] J. Zhang, M. Qiao, Y. Li, Q. Shao, X. Huang. Highly active and selective electrocatalytic CO2 conversion enabled by core/shell Ag/(amorphous-Sn (IV)) nanostructures with tunable shell thickness. ACS Appl. Mater. Interfaces. 11 (43) (2019) 39722-39727.

https://doi.org/10.1021/acsami.9b09092.

[2] W. Ma, S. Xie, X.G. Zhang, F. Sun, J. Kang, Z. Jiang, Q. Zhang, D.Y. Wu, Y. Wang. Promoting electrocatalytic CO2 reduction to formate via sulfur-boosting water activation on indium surfaces. Nat. Commun. 10 (1) (2019) 892.

https://doi.org/10.1038/s41467-019-08805-x.
